# FK228 Analogues Induce Fetal Hemoglobin in Human Erythroid Progenitors

**DOI:** 10.1155/2012/428137

**Published:** 2012-05-14

**Authors:** Levi Makala, Salvatore Di Maro, Tzu-Fang Lou, Sharanya Sivanand, Jung-Mo Ahn, Betty S. Pace

**Affiliations:** ^1^Department of Pediatrics, Georgia Health Sciences University, Augusta, GA 30912, USA; ^2^Department of Chemistry, University of Texas at Dallas, Richardson, TX 75083, USA; ^3^Department of Pharmacological and Toxicological Chemistry, University of Naples Federico II, 80100 Naples, Italy; ^4^Department of Molecular and Cell Biology, University of Texas at Dallas, TX 75080, USA; ^5^Department of Developmental Biology, University of Texas Southwestern Medical Center, Dallas, TX 75390, USA

## Abstract

Fetal hemoglobin (HbF) improves the clinical severity of sickle cell disease (SCD), therefore, research to identify HbF-inducing agents for treatment purposes is desirable. The focus of our study is to investigate the ability of FK228 analogues to induce HbF using a novel KU812 dual-luciferase reporter system. Molecular modeling studies showed that the structure of twenty FK228 analogues with isosteric substitutions did not disturb the global structure of the molecule. Using the dual-luciferase system, a subgroup of FK228 analogues was shown to be inducers of HbF at nanomolar concentrations. To determine the physiological relevance of these compounds, studies in primary erythroid progenitors confirmed that JMA26 and JMA33 activated HbF synthesis at levels comparable to FK228 with low cellular toxicity. These data support our lead compounds as potential therapeutic agents for further development in the treatment of SCD.

## 1. Introduction

Several classes of pharmacological compounds that reactivate *γ*-globin gene transcription have been identified. They include cytotoxic agents, DNA methyl transferase, and histone deacetylase (HDAC) inhibitors. Cytotoxic compounds terminate actively cycling progenitors and perturb cellular growth to trigger rapid erythroid regeneration and *γ*-globin gene activation. S-stage cytotoxic drugs, such as cytosine arabinoside [1], myleran [2], vinblastine [3], and hydroxyurea [4, 5], induce HbF production in primates and humans [4, 6, 7]. The Multicenter Study of Hydroxyurea established this agent as the first FDA-approved treatment for SCD [7]. Hydroxyurea was shown to reduce vaso-occlusive episodes in the majority of sickle-cell patients treated. However, limitations to using hydroxyurea such as bone marrow suppression [8], concerns over long-term carcinogenic complications, and a 30% non-response rate [7, 9], make the development of alternative therapies desirable.

The HDAC inhibitors have also been shown to be potent HbF inducers. These agents target HDACs, which play a dynamic role in regulating cell cycle progression and chromatin conformation by changes in histone acetylation status. Aberrant transcriptional repression mediated by Class I and II HDACs has been demonstrated in many cancers [10]. Thus, HDAC inhibitors have been developed as promising anticancer therapeutics [11]. Structurally diverse classes of natural and synthetic HDAC inhibitors bind target HDACs to block histone deacetylation [12] and produce an open chromatin confirmation and gene activation [13].

There has been great interest in HDAC inhibitors as HbF inducers to treat SCD. They include (1) short-chain fatty acids such as sodium butyrate (NaB), the first HDAC inhibitor reported [14, 15]; (2) the benzamides (i.e., MS-275); (3) non cyclic and cyclic hydroxamates, like SAHA (suberoylanilide hydroxamic acid) and TSA (Trichostatin A); (4) cyclic peptides including FK228 (depsipeptide). NaB induces differentiation in mouse erythroleukemia cells via Stat5 phosphorylation and HbF synthesis through p38 mitogen-activated protein kinase signaling [16–18]. Other fatty acids including phenylacetate and propionate [19–21], induce HbF in erythroid progenitors, however, these agents are rapidly metabolized and oral preparations are not available. These published studies serve as the basis for research efforts to develop HDAC inhibitors as therapeutic agents for SCD.

Of the hydroxamic acid derivatives, the prototype TSA is a potent HDAC inhibitor [22, 23]. It interacts with a divalent zinc-binding motif in the binding pocket of Class I and II HDACs [24]. Other HDAC inhibitors in the hydroxamic acid class include the second-generation analogues of TSA, identified from a library screen of 600 synthesized compounds [25]. The most widely studied TSA analogues are SAHA and Scriptaid. SAHA targets HDAC1, 3, and 4 and inhibits prostate cancer cell growth *in vitro* and *in vivo* [26, 27]. Recently, it was demonstrated by Pace and colleagues that SAHA and Scriptaid induce HbF synthesis comparable to NaB and TSA in erythroid cells and *β*-YAC transgenic mice respectively [28]. However, limitations to the further development of these agents included toxicity in primary cells.

 Another potent HDAC inhibitor is FK228, also known as depsipeptide, isolated from *Chromobacterium violaceum* [29]. This compound has a unique bicyclic structure and is a stable pro-drug activated by the reduction of the disulfide bond by glutathione to produce an active form (redFK) after uptake into cells [30]. The reduced sulfhydryl group interacts strongly with the zinc ion at the active site of the enzyme and has been shown to inhibit tumor proliferation *in vitro* and *in vivo* at nanomolar concentrations [27, 31, 32]. Recently, FK228 was tested in the *μ*LCR*β*_pr_R_luc_^A^ *γ*_pr_F_luc_ GM979 stable cell line and erythroid progenitors grown in methylcellulose colonies produced from peripheral blood mononuclear cells [33]. FK228 was shown to induce HbF in both systems. The level of *γ*-globin and *β*-globin promoter activity was quantified indirectly using firefly (*γ*) and renilla (*β*) luciferase activity.

Drug-mediated HbF induction remains the best approach to ameliorate the symptoms and complications of SCD. Among many compounds, FK228 showed efficacy in inducing *γ*-globin transcription at low concentrations, however, cell toxicity was observed and the drug is difficult to synthesize. It is a bicyclic depsipeptide almost exclusively comprised of unnatural amino acids, D-valine, D-cysteine and, (Z)-dehydrobutyrine (Dhb) as well as a (3S, 4E)-3-hydroxy-7-mercapto-4-heptenoic acid, which is a key component to form the highly constrained bicyclic structure. The high content of the unnatural amino acids and the constrained bicyclic structure make it extremely stable in physiological condition. Simon and coworkers first reported its total synthesis in 1996 [34], and suggested a laborious synthetic route with moderate yield (18% overall yield with over 16 steps).

 Despite its exceptionally high *in vitro* and *in vivo* activity, FK228 has not been explored due to its non-trivial and challenging synthesis, which hampered its production and the design of analogues. The latter would aide our understanding of the molecular mechanism of FK228 and to achieve higher potency and selectivity for HbF induction. In fact, only a few FK228 analogues have been created to date even after intensive synthetic efforts were made [34–36]. To circumvent this problem, we used *in silico *structure analysis and molecular modeling to design twenty FK228 structural analogues that can be easily synthesized [37]. Furthermore, two isosteric substitutions were made without altering its global conformation.

The objective of our study was to investigate the ability of the newly synthesized FK228 analogues to induce *γ*-globin gene transcription using a dual luciferase-based assay system. We identified two lead compounds, JMA26 and JMA33, which induce HbF expression in primary erythroid progenitors. The potential of HDAC enzymes as druggable targets in the treatment of SCD is discussed.

## 2. Materials and Methods

### 2.1. Synthesis of FK228 Analogues

**Scheme 1:**
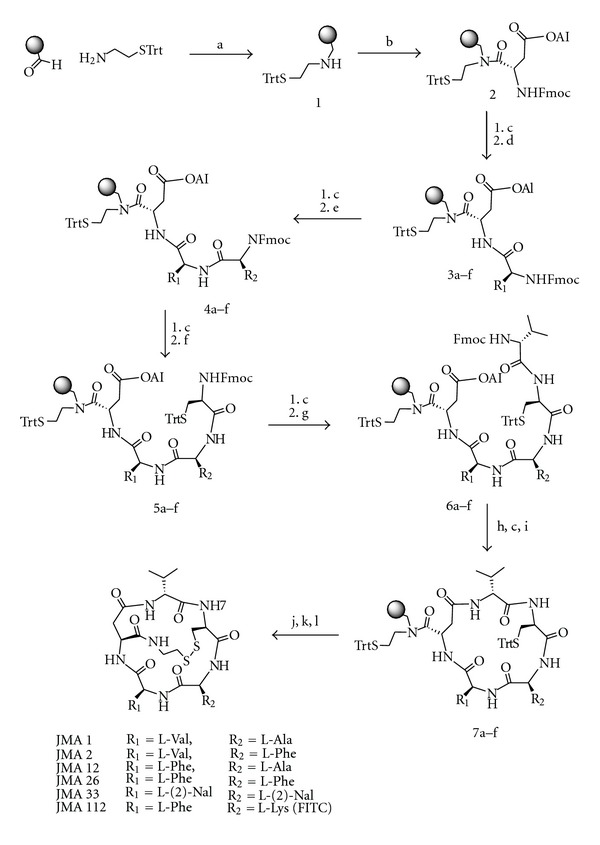
Synthesis of FK228 analogues. Shown in the schematic are the steps, reagents, and conditions used for FK228 analogue synthesis. Compounds 1–7 are the intermediates during the synthesis. For steps 7a–f, the different R_1_ and R_2_ group substitutions were made to generate the various JMA analogues shown. Symbols: (a) NaBH_3_CN; (b) Fmoc-Asp(OAl), DIC; (c) Piperidine; (d) Fmoc-AA_1_, HBTU; (e) Fmoc-AA_2_, HBTU; (f) Fmoc-D-Cys(Trt), HBTU; (g) Fmoc-D-Val, HBTU; (h) Pd(PPh_3_)_4_, DMBA; (i) HBTU; (j) 1% TFA; (k) I_2_; (l) TFA (>95%).

The synthesis of all FK228 analogues described in this study was accomplished by following the previously reported solid-phase synthetic procedure outlined in Scheme 1 [37]. Briefly, S-trityl cysteamine was loaded on aminomethylated polystyrene (AM-PS) resin that was previously functionalized with a backbone amide linker (BAL) [38]. The resulting secondary amine 1 was then coupled with the first amino acid, Fmoc-L-Asp(OAl) to give the aspartylcysteamine 2 with high yield (98%). Thus, the aspartylcysteamine moiety designed to replace the heptenoic acid in the native FK228, was constructed in a single reaction step. The remaining four amino acids were introduced sequentially using the standard N-Fmoc/^t^Bu solid-phase peptide synthesis strategy to build the linear pentapeptides 6a-f. After the allyl and N-Fmoc protecting groups were removed, the macrolactams 7a-f were formed with HBTU (O-benzotriazole-N,N,N′,N′-tetramethyluronium hexafluorophosphate) as a coupling reagent with high yields and purity (>95%). S-Trityl protecting groups were removed with dilute 1% trifluoroacetic acid and the resulting free thiols were oxidized with iodine to produce the bicyclic FK228 analogues. The analogues were cleaved from the resin with TFA (>95%) and characterized by reverse phase—high performance liquid chromatography and electrospray ionization mass spectrometry. 

### 2.2. Cell Culture

Human KU812 leukemia cells were grown in Iscove's Modified Dulbecco's medium (IMDM) (Invitrogen, Carlsbad, CA) with 10% fetal bovine serum (Atlanta Biologicals, Atlanta, GA), penicillin (100 U/mL), and streptomycin (0.1 mg/mL). The cells were incubated at 37°C with 5% CO_2_. Cell count and viability were determined using a hemocytometer and 2% Trypan blue exclusion. Inductions were performed with one million cells treated for 48 hr with the following drugs purchased from Sigma (St Louis, MO): 50 *μ*M Hem (hemin), 2 mM NaB (sodium buytrate), 0.5 *μ*M TSA, 10 mM Cys (cysteine), 1.5 nM FK228, and 100 *μ*M HU (hydroxyurea). We also tested 5 *μ*M SAHA, a gift from Merck & Co. Inc. (Whitehouse Station, NJ).

### 2.3. KU812 Stable Lines

KU812 stable cell lines were created by co-transfecting wild-type KU812 cells with p*EGFP-NI *(G418 selectable marker) *and theμ*LCR*β*_pr_R_luc_^A^ *γ*_pr_F_luc_ dual-reporter a kind gifts from Dr. George Stamatoyannopoulos (University of Washington). Briefly, the 315-bp human *β*-globin gene promoter sequence was inserted upstream of the Renilla along with a polyadenylation signal downstream to create P*β*_pr_R_luc_. Likewise, 1.4 kb of human A*γ*-globin promoter was inserted upstream of firefly luciferase to create ^A^ *γ*_pr_F_luc_. The *μ*LCR (locus control region), P*β*_pr_R_luc_, and ^A^ *γ*_pr_F_luc_ fragments were subsequently cloned into the mammalian vector, pRL-null [39].

The dual-luciferase reporter lines were produced using 10 *μ*g each of linearized *μ*LCR*β*_ pr_R_luc_^A^ *γ*_pr_F_luc_ and p*EGFP-NI *plasmids co-transfected into KU812 cells by electroporation at 260 V, 975 *μ*F (Bio-Rad, Hercules CA). After 72 hr, G418 was added at a concentration of 900 *μ*g/*μ*l for 3 days then maintained under selection pressure indefinitely at a concentration of 400 *μ*g/*μ*l. KU812 stable lines were treated with the various drugs at the same concentrations described above. FK228 and analogues were screened at concentrations between 1–1000 nM for 48 hr and cell toxicity was monitored by 2% Trypan blue exclusion. The effect of drug treatments on *γ*-globin and *β*-globin promoter activity was monitored by luciferase assay.

### 2.4. Dual Luciferase Assay

 Luciferase activity was monitored under the different experimental conditions using the Dual Luciferase Assay Reporter System (Promega, Madison, WI). The activity of firefly luciferase represents *γ*-globin promoter activity (*γ*F), while the renilla luciferase is the read-out for *β*-globin promoter activity (*β*R). The *β*-globin promoter was strategically cloned between the LCR and *γ*-globin promoter to increase *β* expression, while simultaneously increasing the sensitivity of detection of *γ*-globin gene inducers [40].

After drug treatments, KU812 stable cells were washed with 1X phosphate buffered saline and lysed in 1X Passive Lysis Buffer for 15 min, then protein extracts were added to the Luciferase Assay Reagent II and firefly luciferase activity quantified in a Turner Designs TD-20/20 luminometer (Sunnyvale, CA). To measure *β*R activity, Stop & Glo Reagents was added to measure the renilla luciferase activity. Total protein was determined by Bradford assay on a Beckman DU 640 spectrophotometer (Chaska, MN) and luciferase activity was corrected for total protein.

### 2.5. Two-Phase Erythroid Liquid Culture System

 The two-phase liquid culture system was established as previously published by Fibach et al. [41] using buffy coat mononuclear cells, purchased from Carter Blood Care (Fort Worth, TX) in accordance with the guidelines of the Institutional Review Board at the University of Texas at Dallas. During phase 1, cells were grown in IMDM medium with 30% fetal bovine serum and 50 ng/mL each of the granulocyte-monocyte colony-stimulating factor, Interleukin-3, and stem cell factor. Subsequently, phase 2 was initiated on day 7 with the addition of erythropoietin (2 U/mL) and stem cell factor (50 ng/mL). The cells were treated on Day 11 with the different test compounds and harvested on Day 14 (72 hr incubation).

### 2.6. Reverse Transcription—Quantitative PCR (RT-qPCR) Analysis

Total RNA was isolated from samples using RNA Stat-60 (TEL-TEST “B” Inc., Friendswood, TX) and used for RT-qPCR analysis as previously published [42]. Briefly, cDNA was prepared using the Improm-II reverse transcriptase system and oligo (dT)_15 _ primers (Promega). qPCR was performed on an iCycler iQ machine (Bio-Rad) using a master mix containing Sybergreen iQ Supermix (BioRad) and 100 pM of each gene-specific primer pairs for *γ*-globin, *β*-globin and the internal control GAPDH. Standard curves were generated using serial 10-fold dilutions of Topo7 base plasmids carrying a *γ*-globin cDNA sequence (Topo7-*γ*-globin), Topo7-*β*-globin, and Topo7-GAPDH. The globin mRNA levels were calculated as a ratio of GAPDH (*γ*/GAPDH, *β*/GAPDH), and the *γ*/*β*-globin mRNA ratio was calculated by dividing *γ*/GAPDH by *β*/GAPDH.

### 2.7. Immunohistochemistry

Primary erythroid progenitor cytospin cell preparations were fixed with 4% paraformaldehyde in phosphate buffered saline for 15 min, washed, and then permeabilized with 0.3% Trition-100 solution for 10 min. Cells were then blocked in a 5% bovine serum albumin solution and immunostaining performed at 4°C overnight with anti-HbF fluorescein isothiocyanate (FITC) conjugated antibody (Bethyl Laboratories Inc., TX). Cell nuclei were stained with mounting medium containing 4′,6-diamidino-2-phenylindole (DAPI; Santa Cruz Biotechnology, CA). Primary cells were photographed with an Olympus BX 51 phase contrast epifluorescent microscope equipped with Hoffman Modulation optics. Phase-contrast images were recorded with a CCD camera (1/100 sec exposure) and fluorescence images were photographed through 485/520 nm emission filter. The percent of HbF positive cells was calculated by dividing the number of FITC positive cells by total cells (DAPI positive).

### 2.8. Enzyme-Linked Immunosorbent Assay (ELISA)

Total hemoglobin was quantified using 20 *μ*L of protein extract from one million KU812 cells mixed with 5 mL of Drabkin's reagent (Sigma); then cyanmethemoglobin was measured at the 540 nm wavelength. HbF levels were quantified using the human Hemoglobin F ELISA Quantitation Kit (Bethyl Laboratory, Montgomery, TX). Briefly, 96-well plates were coated with sheep anti-human HbF antibody (1 mg/mL). After blocking with 1% bovine serum albumin, horse radish peroxidase-conjugated secondary antibody (1 mg/mL) was added. Raw data were analyzed using GraphPad PRISM (GraphPad Software, Inc., La Jolla, CA) and HbF levels were calculated as a ratio of total hemoglobin corrected for total protein (HbF/total Hb/total protein).

### 2.9. HDAC Inhibition Assay

The HDAC Fluorescent Activity Assay (Enzo Life Science, Farmingdale, NY) was used to measure HDAC inhibition in HeLa cells in a 96-well format using Trichostatin A as the positive control per the manufacturer's protocol. This assay is based on the Fluor de Lys (Fluorogenic Histone Deacetylase Lysyl (FDL)) Substrate/Developer. The procedure was as follows: the test compounds FK228, JMA26, and JMA33 were added to HeLa cells along with the FDL substrate to allow intracellular drug activation then fluorescence levels were read on the fluorometer at 440 nm (CytoFluor II, PerSeptive Biosystems, Farmingdale, NY). The drug concentrations tested were based on the amount required for HbF induction in primary cells. Data was reported as the mean ± standard deviation (SD) for at least five replicates.

### 2.10. Statistical Analysis

The data are reported as the mean ± standard error of the mean (SEM) from at least five data points generated from independent drug treatments. Data were analyzed by a two-tailed student's *t*-test, and values of *P* < 0.05 were considered statistically significant. Statistical analyses were performed using Microsoft Excel (Redmond, WA, USA).

## 3. Results

### 3.1. Isosteric Substitutions Do Not Alter the Global Structure of FK228

**Figure 1:**
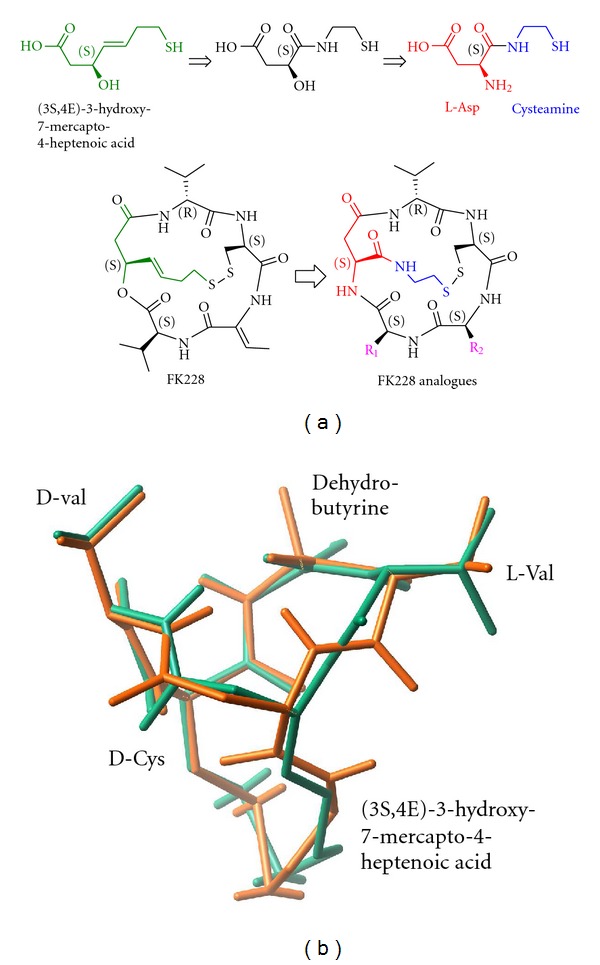
Structures of FK228 analogues. (a) The parent compound FK228 was transformed into novel structural analogues by two isosteric substitutions. The modification of the trans-double bond and ester linkage in the native FK228 with two isosteric amide functional groups allows facile synthesis of analogues as well as retention of the same backbone structure. Various amino acids such as Val, Ala, Phe, 2-Nal, and Lys were introduced to investigate potency of the analogues. (b) Superimposed structures of FK228 (green) and a modified FK228 analogue (orange).

 For the facile synthesis of FK228 analogues, the most synthetically challenging moiety, hydroxy-mercapto-heptenoic acid was modified to a structure that can be easily constructed but has the capability of retaining the structure required for biological activity. We used *in silico *structure analysis and molecular modeling to design structural analogues of FK228 that met these requirements and could be easily synthesized [37]. The design of a novel FK228 analogue is summarized in Figure 1. These compounds were synthesized by modifying the most synthetically challenging unit, (3*S*,4*E*)-3-hydroxy-7-mercaptoheptenoic acid, with two isosteric substitutions without altering its global conformation compared to native FK228. First, the trans-double bond in the heptenoic acid was replaced by an isosteric amide bond. Second, the ester bond required to form the depsipeptide was replaced by another amide bond for facile ring closure that provided higher synthetic yield and increased *in vivo* stability. As shown in Figure 1(b), the structure of the FK228 analogue was found to be almost identical in structure (RMSD = 0.20 Å), indicating that the two isosteric changes neither disturbed the global structure nor altered the backbone structure compared to FK228. However, these changes enabled facile and rapid synthesis using readily available starting materials and high-yielding reactions. While retaining the original stereochemical configurations, the functional groups R_1_ and R_2_ (Figure 1(b)) were substituted with a variety of amino acids such as Ala, Leu, Phe, 2-Nal, Thr, Asp, and Lys (Scheme 1) to examine the side chain consisting of small, large, aromatic, hydrophilic, and charged alterations.

Twenty FK228 analogues were prepared with high overall yield (75–90%) and purity (80–94%) using the solid-phase synthetic strategy. To further characterize the compounds, selected FK228 analogues were examined by 2D-NMR spectroscopy in (dimethyl sulfoxide) DMSOd_6_ using Double-quantum filtered, total correlation, and rotating frame Overhauser effect spectroscopy to confirm structures and stereochemistry (data not shown). The FK228 analogues were shown to have outstanding solubility (10 mM) in the organic solvents ethanol and DMSO and were stable for over one year.

### 3.2. FK228 Analogues Are Potent Inducers of *γ*-Globin Expression

**Figure 2:**
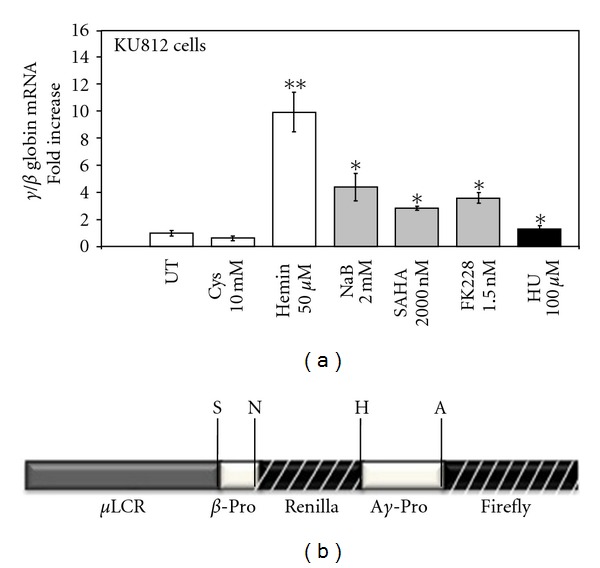
Known HbF inducers activate *γ*-globin expression in KU812 cells. (a) Induction of *γ*-globin transcription by known HbF inducers. Cells were treated for 48 hr with the different drug inducers and *γ*-globin and *β*-globin gene transcription were measured by RT-qPCR (see Section 2). The relative mRNA levels were plotted as fold increase. Untreated cells (UT) were used as a control and normalized to one. Data were calculated as the means ± standard error of the mean (SEM); *P < 0.05 and **P < 0.01. (b) Shown is a schematic of the dual luciferase reporter construct *μ*LCR*β*_pr_R_luc_ *γ*_pr_F_luc_ which contains a 3.1-kb *μ*LCR cassette linked to a 315-bp human *β*-globin gene promoter driving the renilla luciferase gene and a 1.4-kb A*γ* promoter driving the firefly luciferase gene [33, 39].

We first performed drug induction studies in wild type KU812 cells to determine the ability of analogues to induce endogenous *γ*-globin gene transcription. KU812 cells have been classified as a multipotential leukemia cell line with the ability to differentiate down the basophilic [43, 44], eosinophilic [45] and erythroid/megakaryocytic lineages [46]. Previous studies from our laboratory demonstrated that KU812 cells express *γ*-globin, *β*-globin and the erythroid markers CD36, and erythropoietin receptor [47]. Therefore, we used these cells to perform initial drug screens to determine the suitability of KU812 cells for our dual luciferase reporter stable lines. We observed a 1.5- to-10-fold increase in the *γ*/*β*-globin mRNA levels after Hem (50 *μ*M), NaB (2 mM), SAHA (2 *μ*M)), and FK228 (1.5 nM) treatment (Figure 2(a)). In the untreated and negative controls, cysteine-treated cells, *γ*-globin gene expression was not induced. These data demonstrated that the intracellular environment in KU812 is conducive to identifying *γ*-globin gene activators in our FK228 analogue drug screen.

Subsequently, three independent dual-luciferase reporter KU812 stable cell lines were established to analyze the ability FK228 analogues to induce *γ*-globin promoter activity without an effect on *β*-globin transcription. The stable cell lines were created with the *μ*LCR*β*_pr_R_luc_ *γ*_pr_F_luc_ construct (Figure 2(b)) containing a 3.1-kb *μ*LCR cassette linked to a 315-bp human *β*-globin promoter driving the renilla (R) and a 1.4-kb A*γ*-globin promoter driving the firefly (F) luciferase genes [33, 39]. Since the firefly luciferase gene (*γ*F) has approximately 50% greater luminescence than the renilla gene (*β*R), the renilla activity was multiplied by two to adjust for the difference in luminescence [33] yielding the *γ*/*γ*+2*β* final measurement. The FK228 analogues were examined at concentrations ranging from 1–1000 nM in the three stable lines. After 48-hour treatments, cells were harvested and protein isolated for luciferase activity using the Dual Luciferase Reporter Assay. Of the twenty compounds tested, five induced *γ*-promoter activity. The remaining agents were either toxic at the concentrations tested or did not induce *γ*-globin (data not shown).  Table 1 summarizes the *γ*-promoter activity for FK228 analogues that were tested further in primary erythroid cells. Cell viability by Trypan blue exclusion remained at 90–95% for the concentrations shown. Of note are the FK228 analogues, JMA26 and JMA33 (Table 2) containing aromatic side chains in the functional R_1_ and R_2_ groups which produced statistically significant *γ*-promoter activation comparable to FK228. Additional analogues can be designed based on these observations to increase potency, while sparing toxicity.

### 3.3. FK228 Analogues Activate HbF Synthesis in Primary Erythroid Progenitors

**Figure 3:**
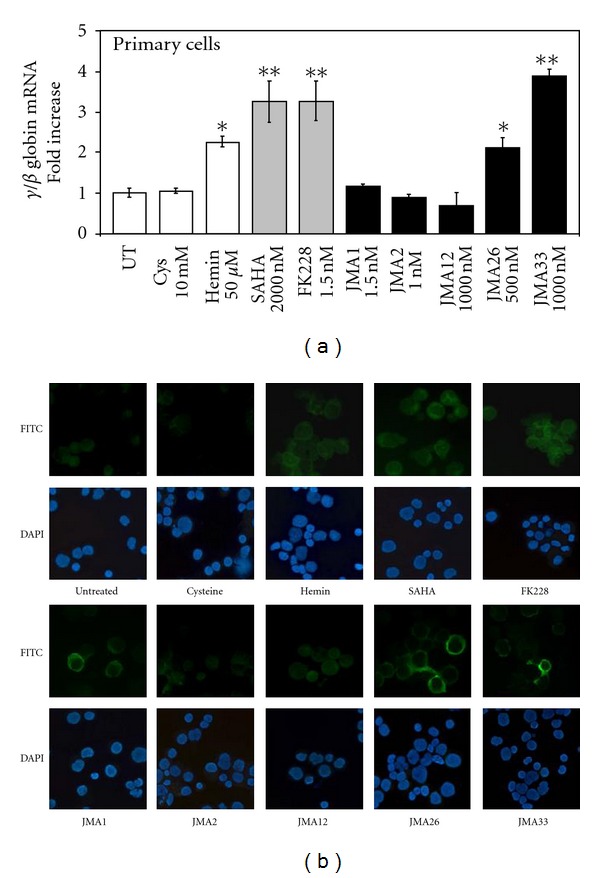
JMA33 and JMA26 induce HbF in primary erythroid cells. (a) Day 11 erythroid progenitors were treated for 48 hr with the controls agents and the FK228 analogues shown and then analyzed by RT-qPCR. Untreated cells (UT) were used as a control and normalized to one. Data were calculated as the means ± SEM. (b) Progenitors were stained with anti-*γ*-globin FITC conjugated antibody overnight and HbF positive cells for were visualized. DAPI staining was performed to identify cell nuclei and to determine cell counts. Images were photographed at 40X power. The images generated were used to count 500 DAPI positive cells and the %FITC positive cells were calculated accordingly.

Next, we examined the ability of the lead FK228 analogues to induce HbF expression in primary erythroid progenitors grown from peripheral blood mononuclear cells in the two-phase liquid culture system. As shown in Figure 3(a), JMA26 and JMA33 induced *γ*-globin transcription at the mRNA level 2.1-fold and 3.9-fold, respectively, compared to a maximal 3.2-fold, induction by SAHA and FK228. However, FK228 derivatives induce *γ*-promoter activity at significantly lower drug concentrations compared to SAHA and Hem. At the concentrations tested, greater than 90% cell viability was observed in primary cells at all concentrations tested for the synthesized compounds. The similarity of these results to those acquired with the KU812 dual-luciferase reporter cell lines also validates the system for drug screening.

The next set of studies was performed to determine the ability of JMA26 and JMA33 to induce HbF in primary erythroid progenitors. Using anti-HbF fluorescein isothiocyanate (FITC) antibody, we observed 15.5% HbF-positive progenitors at baseline in untreated cells (Figure 3(b)). Treatment with JMA26 and JMA33 produced 3.0-fold, and 2.5-fold increase in HbF-positive cells, respectively. A similar increase in HbF-positive cells, were produced by hemin, SAHA and FK228 (3.0-fold, 3.2-fold and 3.5-fold). Complementary ELISA data (Table 3) showed a 1.9-fold and 2.5-fold increase in HbF levels produced by JMA26 and JMA33, respectively, compared to a 2.4-fold HbF induction by FK228. We concluded that these lead compounds have the capability to induce HbF in physiologically normal primary erythroid progenitors.

### 3.4. JMA26 and JMA33 Exhibit HDAC Inhibition Activity

**Figure 4:**
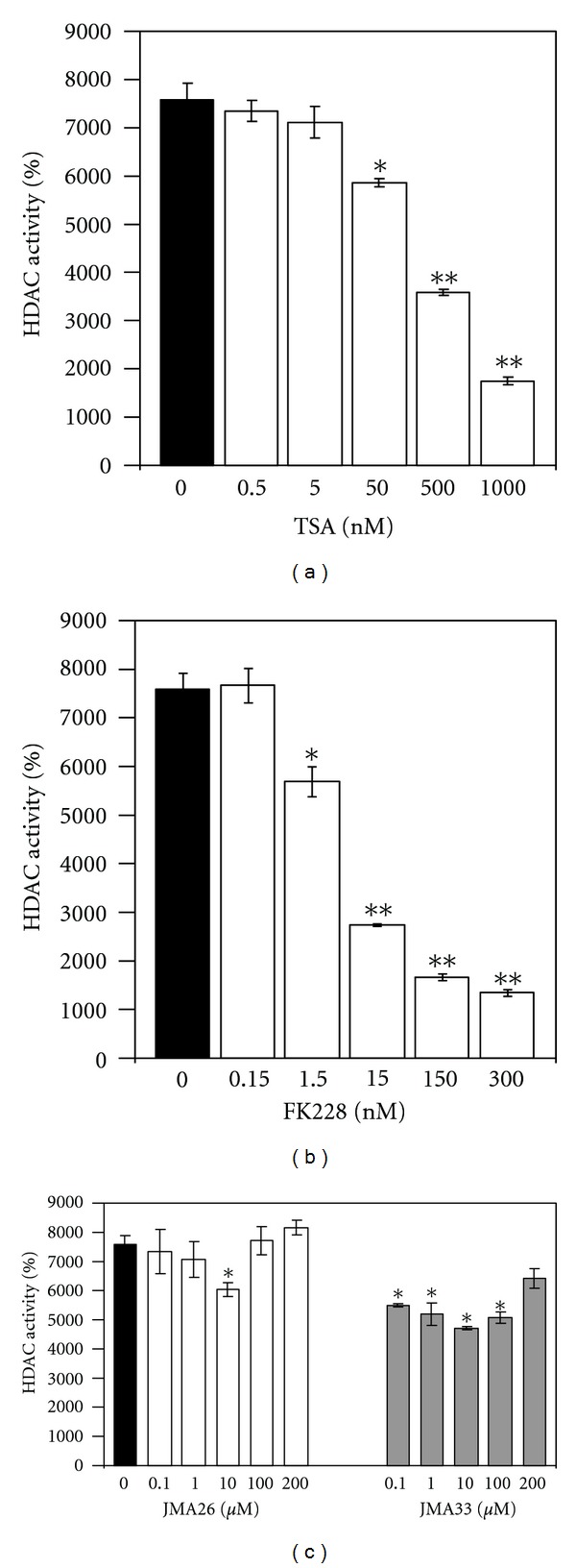
HDAC inhibition assay. The HDAC Fluorescent Activity Assay was used to measure HDAC inhibition in HeLa cells (See Section 2). Compounds and FDL substrate were added to HeLa cells to allow drug activation *in vivo* then fluorescence levels were quantified on the fluorometer at 440 nm. (a) Trichostatin A (TSA) was used as the control. (b) Inhibition studies were conducted for FK228 showing maximal HDAC inhibition at the 300 nM concentration. (c) Similar studies were performed for JMA33 and JMA26. The data are reported as the means ± SEM.

To ascertain the mechanism of HbF induction by the lead compounds, we performed an *in vivo* assay to investigate the ability of JMA26 and JMA33 to act as HDAC inhibitors. The HDAC Fluorescent Activity Assay designed to measure HDAC activity in HeLa cells was completed in a 96-well format. The assay is based on the fact that the Fluor de Lys substrate is deacetylated by HDACs to generate a fluorescent readout. TSA (0.5 to 1000 nM) was used to establish the assay in HeLa cells, showing about 80% HDAC inhibition in our system (Figure 4(a)). By contrast, FK228 produced about 85% inhibition at the 300 nM concentration, which produces marked cell toxicity (Figure 4(b)). Similar studies performed for JMA26 and JMA33 showed 20% and 37% HDAC inhibition, respectively (Figure 4(c)), suggesting HbF induction in erythroid cells occurs by other mechanisms.

## 4. Discussion

Drug-mediated HbF induction remains the best treatment approach to ameliorate the symptoms and complications of SCD due to its ability to inhibit hemoglobin S polymerization. In addition, HbF provides an effective treatment for *β*-thalassemia by correcting globin chain imbalance [48]. Other therapies aimed at the underlying molecular causes of the *β*-hemoglobinopathies include hematopoietic stem cell transplantation [49] and gene therapy involving the transfer of normal *γ*- or *β*-globin genes into hematopoietic stem cells. Despite promising results and ongoing research, the option for stem cell transplantation is limited by the lack of suitable donors for the majority of SCD patients. On the other hand, gene therapy offers a universal cure but there are concerns about mutagenesis of target genes due to random vector integration and the effects of viral sequences on nearby gene expression [50]. Therefore, pharmacologic HbF induction remains a viable choice for the development of additional therapeutic options for treating SCD.

Hydroxyurea is the only drug approved by the Food and Drug Administration for the treatment of SCD [7, 51], however, it is not effective in all patients [7] and of minimal benefit in *β*-thalassemia [52]. Moreover, there are concerns about undesirable side effects including long-term carcinogenesis [53]. Clinical trials with other compounds, such as arginine butyrate [54] and decitabine [55] have shown considerable promise, however, orally active preparations need to be developed to make these agents viable treatment alternatives.

For many years, K562 cells have been used to screen pharmacological agents as potential HbF inducers. For example, NaB, decitabine, and hydroxyurea, among others, stimulate erythroid differentiation in K562 cells and induce *γ*-globin gene transcription [56]. Many HDAC inhibitors including FK228 are also known to induce HbF. However, synthetic difficulties associated with FK228 production have severely deterred structure-activity studies to aid understanding of its mechanism of action and to improve efficacy. Our data shows that JMA26 and JMA33 increased HbF levels by a mechanism independent of HDAC inhibition.

Many published studies have shown that primary erythroid cells remain the best system to confirm HbF-inducing agents and to serve as a predictor of efficacy *in vivo*. Human burst forming units—erythroid cells in clonogenic assays [57] or erythroid progenitors grown in liquid culture [39, 41]—have been used to evaluate putative HbF inducers. However, these assays are not easily adaptable to large-scale drug screening, thus immortalized cell lines have been investigated for this purpose. Previously, FK228 was tested in the *μ*LCR*β*_pr_R_luc_^*A*^ *γ*_pr_F_luc_ GM979 stable line [33] and was shown to induce *γ*-promoter activity at the 1 nM concentration. We expanded on these studies to establish a dual-luciferase reporter system. Thus, we used KU812 cells derived from an individual with chronic myeloid leukemia [58] because both *γ*-globin and *β*-globin are actively transcribed [47]. Moreover, gene profiling data generated by our laboratory showed that KU812 cells express CD36 and the erythropoietin receptor at levels comparable to day-14 human erythroid progenitors [47].

In this study, when wild-type KU812 cells were treated with Hem, NaB, SAHA, and FK228, we observed a 3- to 10-fold increase in the *γ*/*β*-globin ratio. We next tested the FK228 analogues in the KU812 dual-luciferase reporter system created with the *μ*LCR*β*_pr_R_luc_^A^ *γ*_pr_F_luc_ construct. Two FK228 analogues identified in the reporter assay, JMA26 and JMA33, showed efficacy as HbF inducer in primary erythroid progenitors suggesting these compounds have the potential for further development.

Our last set of experiments was aimed at understanding the mechanism by which JMA26 and JMA33 induce *γ*-globin. Histone acetylation is a highly dynamic reversible modification that contributes to gene expression through changes in chromatin conformations. The parent compound FK228 is a class IV cyclic peptide capable of inhibiting Class I HDAC enzymes (HDAC1, 2, 3, and 8) after intracellular reduction of its disulfide bond by glutathione to produce the active reduced form of FK228. The functional sulfhydryl group fits inside the catalytic pocket producing zinc chelation and inhibition of enzymatic activity [59].

The role of HDAC inhibition in HbF induction has been investigated by several laboratories. NaB was the first agent shown to mediate histone H3 and H4 hyperacetylation as a mechanism of HbF induction [60]. Subsequently, many other HDAC inhibitors such as TSA [42], scriptaid [28], SBHA (suberohydroxamic acid), and SAHA (suberoylanilide hydroxamic acid) [59] were shown to be HbF inducers based on the central role of histone hyperacetylation. Subsequently, Perrine and colleagues showed the ability of short-chain fatty acids to induce *γ*-globin by displacement of an HDAC3-NcoR repressor complex [61]. More recently, there exist chemical genetic screen-identified HDAC1 and HDAC2 as molecular targets facilitating drug-mediated HbF induction [62]. Therefore, to determine the mechanism of action of JMA26 and JMA33, we completed the HDAC inhibition assay.

Using the Fluor de Lys system, FK228 produced strong HDAC inhibition but at a higher concentration (300 nM) than required for HbF induction. Similar studies performed for JMA26 and JMA33 showed 20% and 37% maximal inhibition, respectively. These findings suggest that the alterations in FK228 structure may have uncoupled HDAC inhibition activity as the primary mechanism of HbF induction since higher test drug concentrations did not produce more HDAC inhibition. These data suggests JMA26 and JMA33 may induce *γ*-globin by mechanisms other than targeting HDACs. Since the FK228 analogues were developed from a structural library designed by molecular modeling, additional compounds can be synthesized with greater HbF inducing potency and selectivity to Class I HDACs. Additional studies will also be conducted to determine other mechanisms by JMA26 and JMA33 that induce HbF such as activation of the p38 mitogen-activated protein kinase or other signaling pathways [17, 63, 64].

## 5. Conclusions

The current drug treatment options for SCD are limited with hydroxyurea being the only FDA-approved drug. The key finding of this study is the high-efficiency synthesis of FK228 analogues with structural modifications which did not disturb the global chemical structure of the parent compound. The analogues exhibited HbF induction at nanomolar concentrations in primary erythroid progenitors demonstrating physiological relevance. These data support the FK228 analogues as potential therapeutic agents and also validates the KU812 dual-luciferase stable cell lines as an efficacious screening system to identify *γ*-globin activators. Long-term our goal is to establish a group of HbF inducers that selectively inhibit Class I HDACs to expand our understanding of epigenetic mechanisms of *γ*-globin gene regulation and to facilitate the development of drug therapy for SCD.

## Figures and Tables

**Table 1:** *γ*-globin induction in KU812-*γ*F/*β*R stable lines by FK228 derivatives^1^.

Line 1	Drug concentration	*γ*/*γ* + 2*β* Mean	SEM	*P* value	Fold change
Untreated	none	0.0402	0.0091	n/a	1
Cys	10 mM	0.0388	0.0092	0.8528	0.950
Hemin	50 *μ*M	0.1732	0.0399	0.0050	4.325
SAHA	2000 nM	0.2157	0.0142	0.0001	5.400
FK228	1.5 nM	0.1197	0.0325	0.0150	2.975
1127Ox	1000 nM	0.0363	0.0022	0.8167	0.900
JMA1	1.5 nM	0.0473	0.0149	0.6720	1.175
JMA2	1.0 nM	0.0157	0.0003	0.1629	0.400
JMA12	1000 nM	0.0187	0.0009	0.2156	0.475
JMA26	500 nM	0.1460	0.0234	0.0004	3.650
JMA33	1000 nM	0.1373	0.0117	0.0002	3.425
JMA112	100 nM	0.0187	0.0007	0.2156	2.453

Line 2	Drug concentration	*γ*/*γ* + 2*β* Mean	SEM	*P* value	Fold change

Untreated	none	0.1573	0.0150	n/a	1
Cys	10 mM	0.1200	0.0056	0.1977	0.764
Hemin	50 *μ*M	0.6593	0.0839	0.0001	4.261
SAHA	2000 nM	0.6500	0.0208	0.0001	4.140
FK228	1.5 nM	0.6167	0.0338	0.0001	3.929
1127Ox	1000 nM	0.6133	0.0371	0.0001	3.904
JMA1	1.5 nM	0.1260	0.0152	0.2896	0.803
JMA2	1.0 nM	0.1183	0.0071	0.1814	0.752
JMA12	1000 nM	0.1470	0.0027	0.7093	0.936
JMA26	500 nM	0.2487	0.0308	0.0153	1.579
JMA33	1000 nM	0.3313	0.0256	0.0002	2.108
JMA112	100 nM	0.1070	0.0076	0.0938	0.682

Line 3	Drug concentration	*γ*/*γ* + 2*β* Mean	SEM	*P* value	Fold change

Untreated	none	0.0721	0.0052	n/a	1
Cys	10 mM	0.0860	0.0050	0.1814	1.194
Hemin	50 *μ*M	0.2508	0.0153	0.0001	3.486
SAHA	2000 nM	0.2520	0.0066	0.0001	3.500
FK228	1.5 nM	0.1583	0.0198	0.0007	2.194
1127Ox	1000 nM	0.0370	0.0095	0.0076	0.514
JMA1	1.5 nM	0.0770	0.0036	0.6179	1.069
JMA2	1.0 nM	0.0740	0.0036	0.8463	1.027
JMA12	1000 nM	0.0537	0.0023	0.0779	0.750
JMA26	500 nM	0.2560	0.0192	0.0001	3.555
JMA33	1000 nM	0.5120	0.1765	0.0007	7.111
JMA112	100 nM	0.0467	0.0019	0.0216	0.653

^1^Using three independent KU812-*γ*F/*β*R stable cell lines, the FK228 analogues were examined for their ability to induce *γ*-globin. After 48 hr drug treatments, cells were harvested and dual luciferase assay performed. Untreated cells were used as a control and normalized to one. Data were calculated as the means ±  standard error of the mean (SEM).

**Table 2:** FK228 structural analogues.

FK228 analogues	R_1_	R_2_
JMA1	Val	Ala
JMA2	Val	Phe
JMA12	Phe	Ala
JMA26	Phe	Phe
JMA33	2Nal	2Nal
JMA112	Phe	Lys(FITC)

Val: valine; Phe: phenylalanini; Ala: alanine; 2Nal: 2-naphthylmethyl; Lys: lysine; FITC: fluorescein isothiocyanate.

**Table 3:** Fetal hemoglobin quantification in primary erythroid cells.

	Drug concentration	Mean	SEM	*P* value	Fold change
Untreated	none	0.683	0.0291	n/a	1
Cys	10 mM	0.737	0.1201	0.7773	1.079
Hemin	50 *μ*M	1.515	0.0405	0.0001	2.218
SAHA	2000 nM	1.094	0.1049	0.0197	1.602
FK228	1.5 nM	1.676	0.0506	0.0001	2.454
JMA1	1.5 nM	0.7953	0.0849	0.2803	1.164
JMA2	1.0 nM	0.8120	0.0165	0.0183	1.188
JMA26	500 nM	1.3086	0.0535	0.0005	1.916
JMA33	1000 nM	1.7223	0.0725	0.0002	2.521
